# Breast Cancer-Related Lymphedema Assessed via Tissue Dielectric Constant Measurements

**DOI:** 10.7759/cureus.59261

**Published:** 2024-04-29

**Authors:** Carel Toro, Biura Markarian, Harvey N Mayrovitz

**Affiliations:** 1 Osteopathic Medicine, Nova Southeastern University Dr. Kiran C. Patel College of Osteopathic Medicine, Fort Lauderdale, USA; 2 Medicine, Nova Southeastern University Dr. Kiran C. Patel College of Allopathic Medicine, Davie, USA

**Keywords:** edema, measuring lymphedema, tdc, tissue dielectric constant, lymphedema, bcrl, breast cancer

## Abstract

This review describes the use of tissue dielectric constant (TDC) measurements mainly in the assessment of breast cancer-related lymphedema (BCRL). PubMed, Web of Science, and EMBASE databases were initially searched using criteria that included the terms “dielectric” and “lymphedema.” The initial search yielded a total of 131 titles. After removing studies not focused on upper extremity lymphedema, 56 articles remained. These articles, together with relevant articles from their bibliographies, formed the basis of the review. The findings show the potential utility and applications of TDC measurements to help detect and track BCRL, whether present in limbs, breasts, or trunks. It is reported as a non-invasive, simple-to-use method, with each measurement requiring less than 10 seconds, suggesting its practicality and useability as an in-office or in-clinic screening and tracking method. Although there are various ways to quantitatively evaluate lymphedema, most, if not all, are restricted to measurements on limbs. Thus, one significant advantage of the TDC approach is that almost any local region of interest can be effectively measured and tracked, which, for BCRL, could include specific regions of arms or hands, breasts, and truncal areas.

## Introduction and background

Breast cancer-related lymphedema (BCRL) incidence varies widely, with multiple methods used to identify it [1-5]. Methods include measurements of limb circumference [6-9], limb volume [10-14], and limb electrical impedance [15-19]. Another approach is via the measurement of tissue dielectric constant (TDC) values at 300 MHz, which provides a valuable way to assess edema or lymphedema in a localized region [20]. Pioneering research has led to the development of various techniques to characterize the dielectric properties of biological tissues over a wide range of frequencies [21-24]. Subsequent research and engineering innovations resulted in the creation of a practical probe to measure TDC for research and clinical purposes [25,26]. Currently, TDC is measured via handheld probes containing concentric conductors, serving as open-ended coaxial probes that can be directly applied to the skin [27]. The device's probe emits a low-power electromagnetic signal, taking only a few seconds to measure [27]. By analyzing the reflected signals from different tissues, the device discerns the tissue's dielectric constant, providing a reliable indicator of its water content [27]. The primary objective of this review is to outline and elucidate the application of TDC measurements in evaluating BCRL, serving as a potential screening or tracking tool for lymphedema progression.

## Review

The following outlines the specifics of the study's eligibility criteria and search parameters employed in this review.

Materials and methods

The search initially targeted PubMed, Web of Science, and EMBASE databases using specific criteria incorporating the terms "dielectric" and "lymphedema." This initial search yielded a total of 131 articles from the PubMed search, 91 from Web of Science, and 109 from EMBASE. The Web of Science and EMBASE articles that were duplicates of those within the PubMed search were removed, yielding 131 articles. Subsequently, studies that are not primarily centered on or related to upper extremity lymphedema were excluded, leaving 56 relevant articles. These selected articles, along with additional references sourced from their bibliographies, formed the foundational material for the literature review. The following delineates the various TDC devices and their measurement criteria.

TDC devices

**Figure 1:**
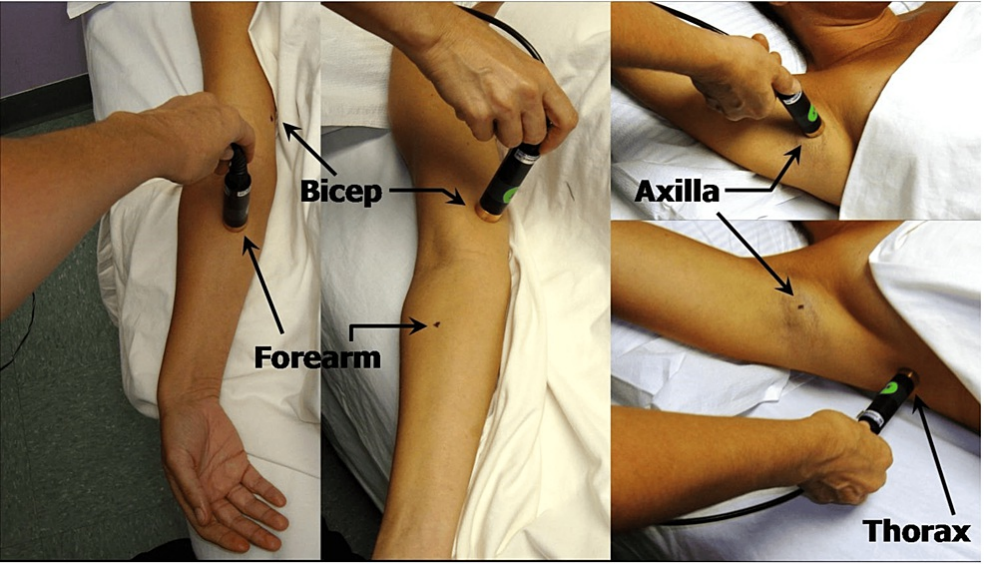
Illustration of TDC measurements Measurements on the forearm, biceps, thorax, and axilla are illustrated using a TDC probe with an effective measurement depth of 2.5 mm. Image credits: This figure was courtesy of Dr. Mayrovitz. TDC: Tissue dielectric constant.

As per existing literature, the characteristics of these probes, including their penetration depth, rely on the size and structure of the probe and have been investigated by various researchers [28-30]. A commercial version emerged (Delfin Technologies, Kuopio Finland) in which four different-sized probes were offered with outside dimensions ranging from 10 to 55 mm with effective skin penetration depths at 300 MHz of 0.5 to 5.0 mm, respectively. Figure 1 demonstrates a probe measuring TDC on the forearm, biceps, thorax, and axilla.

**Figure 2:**
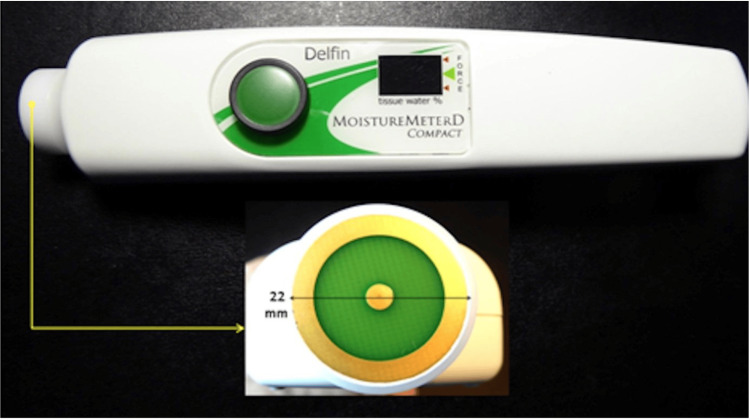
Compact version of the TDC measurement device Signal generation and processing are contained in the handheld device. Image credits: This figure was courtesy of Dr. Mayrovitz. TDC: Tissue dielectric constant.

Although the probe shown in Figure 1 is handheld, it needs to be connected to a small control box that generates the electromagnetic wave that penetrates the skin, processes the reflected wave information, and displays TDC values. Subsequently, a compact version was developed as shown in Figure 2, with a penetration depth between 2.0 and 2.5 mm [31]. Each of the available measurement devices has its own separate pros and cons. The original multiprobe set offers the possibility to evaluate tissues to varying depths ranging from 0.5 to 5.0 mm [32] but is a bit less mobile than the handheld compact version, which could easily fit into a pocket. The compact probe offers the advantage of size and also incorporates a pressure-sensing process that may increase repeatability, but this probe is restricted to a single effective measurement depth [33].

Measurement reliability

TDC test-retest measurements in 40 healthy volunteers done by two investigators were used to determine the minimum detectible change and interclass coefficients [34]. A minimal detectible change that ranged from two to nine TDC units was reported with a corresponding interclass coefficient of 0.765 to 0.982 [34]. Additional findings based on measurements in 30 females who had BCRL supported these findings [35].

Anatomical site dependency

TDC values can vary depending on the anatomical site being measured. Thus, investigations have been done at different locations on the forearm, upper arm, and legs [32,36-38]. An evaluation of 30 healthy females was done, which included TDC measurements on the midline of the forearm and sites medial and lateral to the midline that were four, eight, and 12 cm distal to the antecubital crease [36]. Based on these measurements, it was reported that midline and medial values increased from proximal to distal sites (p < 0.001) [36]. Although variations among sites were reported as being less than 10%, this amount may be important when assessing patients with BCRL. As a consequence, the chosen sites for measurement should be consistent when assessing changes or when comparing groups. This approach is emphasized by findings on these patients with BCRL. Being left- or right-handed does not affect TDC values [39]. In addition to variations longitudinally along the arm, TDC value differences between anterior and medial locations at the same longitudinal site have been reported [40].

Measurement depth dependency

Tissues containing higher levels of fat tend to retain less water, resulting in lower TDC values. This means that in regions of the body where there is more subcutaneous fat, particularly at deeper levels, TDC values are typically lower. This has been demonstrated in several studies [41-44]. An evaluation of 40 healthy women in whom forearm TDC was measured bilaterally and compared to total body water percentage showed a positive correlation between total body water percentage and total body fat percentage when TDC was measured to a depth of 5 mm [40]. When such measurements were done on women who were overweight or obese, a similar depth dependence of TDC was reported [42]. However, not all anatomical sites show a decrease in TDC values with increasing measurement depth. Values obtained with increasing depths from 0.5 to 2.5 mm increased when measured on the palm, thenar eminence, and great toe with little change in depth when measured at the hand and foot dorsum skin [37]. Choosing which depth to measure is ultimately based on probe availability and importantly the intent of the measurement.

Measurement number per site

TDC measurements primarily rely on the water content of the interrogated tissue volume. The size of this examined volume is primarily determined by the diameter of the probe utilized and the effective depth of penetration. The question of whether to use single or multiple measurements at a specific anatomical site has been investigated [45,46]. In one study, the criteria to evaluate this was the agreement between single and averaged triplicate measurements [45]. To answer this question, forearm TDC was determined in 10 females with BCRL. The 95% confidence interval (CI) for differences between single and averaged TDC values was less than ±1 TDC unit. In the other study, TDC values were obtained in 20 healthy females at depths of 0.5-5.0 mm on forearms and lateral thorax as well as also on 10 women with BCRL to a depth of 2.5 mm. The 95% CI for differences between single and averaged TDC values were below ±1 TDC unit, and the limits of agreement between methods were below ±2.5 TDC units (±6.5%). Thus, if this difference is acceptable in the clinic, a single measurement may be adequate. Other studies evaluated different anatomical locations. Single, double, and triplicate measurement outcomes measured on the forearm, palm, lateral, medial calf, and foot dorsum were compared [47]. It was found that the forearm had the smallest coefficient of variation (2.19%), and the lateral calf had the largest value (4.59%) [47]. These results suggest that when there are time pressures in the clinic, one TDC value may suffice for arm measurements, but duplicate or triplicate values are needed for leg assessments.

Time-of-day dependency

Understanding the potential for TDC value fluctuation based on the time of day is beneficial, given that measurements might be taken at various times throughout the day. To investigate the presence of a circadian dependency, TDC was measured every hour from 0800 to 2000 hours in 12 adult females [48]. Measurement sites were the face below the eye, cheek, forearm, and calf. All upper body values were reported to decrease from morning to night from 11.2% to 5.6%, whereas calf values progressively reduced from an average of 9.3% [48]. These findings indicate that time-of-day measurement may be a factor to be considered, but if inter-limb ratios are used, time of day is less important [49].

Age dependency

TDC measurements were used to investigate tissue water distributions in healthy skin and to study possible age and BMI impacts [50]. In that study, forearm TDC values were obtained from 69 healthy females who had a wide range of both age (22-82 years) and BMI (18.7-46.1 kg/m^2^) [50]. TDC values decreased with increasing depth, although inter-arm ratios were similar and close to unity. The age factor was also evaluated in 200 women in whom TDC was measured to varying depths, and it was found that at shallower depths, older women had higher values [51]. These and prior findings suggest an age-related shift in skin water distribution and a change from less to more mobile water states. Similar changes with age were reported in 60 males [52] and 60 females, both of whom had increased TDC only in the shallowest measurement depth of 0.5 mm [53]. The age-related changes may impact long-term longitudinal tracking studies, among others. These values may help formulate age-dependent TDC reference values for TDC.

Gender dependency

Thirty males and 30 females had TDC values measured on forearms, forehead, and cheek to a measurement depth of 1.5 mm [54]. Male values were greater than female values (p < 0.01), with differences of 5.6% at the forehead to 11.3% at the forearm [54]. Gender differences were also reported in a study of 32 males and 32 females, with male values greater than female values at the forearm and biceps [31]. In a study with TDC measurements on the forearms of 280 males and 280 females, a greater TDC value for males was found for all measurement depths [43].

Race dependency

TDC differences to a depth of 1.5 mm in forearms potentially due to race or ethnicity were investigated in 100 persons composed of 20 Caucasians, 20 African-Americans, 20 Asian Indians, 20 Asians, and 20 Hispanics [55]. Results indicated that for females, there was an overall difference among the races, with Caucasians having the highest values [55]. However, forearm TDC values obtained on male subjects did not differ among races [55]. These reported results indicate that race is a factor to consider when absolute TDC values are used.

BCRL evaluations

Initial studies that primarily focused on the use of TDC for BCRL patients were described in 2007 [20]. In that study, bilateral forearm TDC values were obtained in 18 healthy female volunteers and 15 patients with unilateral BCRL [20]. TDC values measured on the patient’s lymphedematous arm were significantly greater than those in the non-affected arm [20]. Furthermore, when TDC values were compared between affected and non-affected arms, an inter-arm TDC ratio (IAR) in patients of 1.64 ± 0.30 was found. But in the healthy group, the IAR was only 1.04 ± 0.04, which was a highly significant difference between groups (p < 0.001) [20]. For the small number of patients that were evaluated, none had an IAR ratio as low as 1.2, and no healthy non-edematous subject had an IAR that was as great as 1.2 [20]. An extension of this work included bilateral TDC measurements in 30 females diagnosed with BCRL, 30 females diagnosed with breast cancer who had not yet had their surgery, and 30 healthy females without a diagnosis of breast cancer [56]. TDC values did not differ between females with or without breast cancer, but for those who had lymphedema, the values were significantly higher [56]. IAR values for the combined non-lymphedematous women were reported as 1.006 ± 0.085 and were significantly less than the patient’s IAR of 1.583 ± 0.292 [56]. Based on these findings, a threshold IAR for lymphedema detection (TDC of at-risk side/TDC of contralateral side) was determined to be 1.26 [56].

Further work in this direction investigated TDC values obtained in three groups of females with 80 participants per group [57]. Considering an IAR threshold equal to three standard deviations from the mean IAR, a threshold value of 1.2 was also indicated [57]. Additional studies of IAR for early detection were done in 100 women who had been treated with breast surgery and had axillary dissection and radiotherapy (BIS) [58]. TDC values indicated that 18.4% of early BCRL were detectible with the TDC method, whereas only 2.6% were detectible with bioimpedance spectroscopy [58]. Furthermore, it was reported that concerning the 38% of patients diagnosed with lymphedema, the TDC method had a detection sensitivity of 65.8% and a specificity of 83.9% [58].

In another study, TDC measurements were made bilaterally on the forearm and biceps of 207 females, with 104 having been previously surgically treated for breast cancer and 103 who had breast cancer but were awaiting surgery [59]. An IAR threshold for lymphedema was determined to be 1.3 for the forearm and 1.45 when measured at the biceps [59]. These IAR thresholds formed the basis of an evaluation of 80 females who were measured before their breast surgery and then for up to two years later. In a further study of 84 women with 42 awaiting breast cancer surgery and 42 healthy volunteers, three SD IAR thresholds of 1.20 and 1.24 for forearm and biceps were determined [60]. A test of the suitability of this IAR threshold was evaluated in 63 females, of whom 32 had BCRL and 31 had breast surgery for cancer but were free of BCRL [61]. The sensitivity and specificity of detecting BCRL based on inter-arm TDC ratios were reported as 65% and 94%, respectively [61]. Other work was directed to determining IAR thresholds for hands in 70 women, half aged less than 35 years and half older than 50 years [62]. Results revealed an age-independent IAR threshold of 1.23. An extension of this work evaluated 120 females who were awaiting breast cancer surgery [63]. In this study, the primary focus was on determining the proposed inter-side ratio (ISR) threshold based on TDC measurements of the lateral thorax [63]. A thorax-to-thorax ISR of 1.32 was reported. In a comparative study between IAR arm thresholds and water displacement, it was discovered that both methods proved beneficial. However, the TDC method, when employed independently, exhibited an ability to diagnose lymphedema at an earlier stage than water displacement [64].

Breast lymphedema

Breast lymphedema is also present in some patients with BCRL and in some as the only complication of breast surgery or other conditions [65]. The use of TDC measurements for breast assessments has been investigated by several groups [66-70]. Using TDC values based on measurement in both breasts of healthy women, an ISR diagnostic threshold of 1.40 was tested for use in 118 women after their breast cancer surgery and radiotherapy [66]. Based on that ISR criterion, 31.4% of patients indicated breast edema following their surgery but before the end of radiotherapy [66]. The percentage of patients exceeding the ISR threshold increased to 62.4% when measurements were done four weeks after radiotherapy was completed [66]. However, when measures were done nearly two years later, on 65 remaining patients, the percentage of patients exceeding the ISR fell to 28% [67]. As may have been anticipated, using the ISR threshold of 1.4 was consistent with the findings in a group of 10 patients with documented breast lymphedema [68]. TDC methods were also of use in characterizing changes in breast edema of 56 breast cancer-treated patients who had breast edema as determined by the 1.40 threshold [69]. TDC IAR has also been used to determine the need to reestablish breast compression to minimize the chance for it to become sustained lymphedema [71]. In addition to breast lymphedema, truncal lymphedema occurring after breast cancer treatment has been examined using TDC techniques [72-75].

Discussion

This review focuses on the characteristics and functionalities of TDC measurements, along with their applications as a tool for screening and monitoring BCRL. Other potential origins of upper extremity lymphedema, such as those arising after melanoma treatment, have not been explicitly discussed. However, the broad utility of TDC remains applicable for the detection and evaluation of lymphedema. Several other potential uses have not been covered in which TDC measurements have been reported for the assessment of lymphedema in other anatomical areas. These areas include the lower extremities [76,77], head and neck [78,79], breast, and trunk. Further, given the planned narrow focus of the present review, other modalities for assessing upper extremity lymphedema have not explicitly been reviewed or compared herein. Each of these modalities has method-specific pros and cons that relate to multiple factors, including the ability to measure local lymphedema, the relative cost of the equipment, the cost of the measurement procedure, the information content that emerges from the assessment, its ease of implementation, and its track record as evidenced by outcomes. For readers interested in following up on these other modalities, critical methods and references include bioimpedance spectroscopy [80,81], magnetic resonance imaging [82], water displacement [83,84], limb volumes [85,86], and lymphoscintigraphy [87,88]. It is also noteworthy that some studies suggest that combining TDC and limb volumes are useful complementary methods to evaluate BCRL [89].

Concerning the main findings of the present review, among the various methods to quantitatively assess lymphedema, TDC measurements are the only well-documented method proper for in-office or in-clinic assessment of almost any anatomical area and not restricted to limb measurements. As specifically applied to assessments of upper extremity lymphedema, its potential advantage lies in part with its ability to measure selective areas of the upper extremity that appear most affected in comparison with measurements made either on the other arm at anatomically similar locations or a standardized non-affected location. Another potential advantage is the reported relative speed with which measurements can be made in office or clinic settings. This feature would suggest that it has significant potential to be used as a screening measurement and also as a tracking measurement on repeated visits. Reviewed studies have indicated that TDC measurements be made before surgery to optimize detection. The short measurement time would facilitate the implementation of this strategy.

The reviewed literature describes measurements made to different depths ranging from about 0.5 to 5.0 mm below the skin surface, with no clear indication of which might be optimum for a given situation. Although measurements to different depths may be revealing, dependent on the measurement purpose, most reported measurements on patients were made to a depth of about 2.5 mm. Unless there is a specific requirement for measuring at a different depth, it is advisable to utilize the most reported depth of 2.5 mm. An exception to this would be if there is a particular interest in assessing fluid at the epidermal and dermal levels, in which case a depth of 0.5 mm might be recommended.

## Conclusions

The results of this review highlight the potential of TDC measurements in detecting and monitoring BCRL, regardless of whether it affects the limbs, breasts, or trunk. Additionally, the findings suggest that this method is non-invasive and user-friendly, with each measurement taking less than 10 seconds once the device is placed on the skin. Moreover, multiple devices are available that enable TDC measurements at various depths, from the epidermis to the subcutaneous fat layer, offering potential advantages for both research and clinical use. Although there are a variety of ways to evaluate lymphedema quantitatively, most of the readily usable and easily available methods, including bioimpedance spectroscopy, are generally restricted to measurements on limbs. Thus, an additional advantage of the TDC method and approach that has been reported and verified is that it can be used in almost any local region of interest to measure and track BCRL effectively.
